# We “R” capable of leading CPD in challenging times: A suggested framework for CPD management during COVID-19 and beyond

**DOI:** 10.15694/mep.2021.000002.1

**Published:** 2021-01-11

**Authors:** Samar Aboulsoud

**Affiliations:** 1 Cairo University

**Keywords:** Continuing professional development, healthcare, COVID-19, medical education, accreditation, regulation

## Abstract

This article was migrated. The article was marked as recommended.

The Coronavirus (COVID-19) outbreak is arguably one of the greatest public health challenges of our time. Health care providers (HCP) play a vital role in helping to treat and contain Coronavirus and should the virus spread further, HCP are likely to face an increased burden in helping contain the outbreak and in supporting patients and families.

This article presents a management framework for continuing professional development (CPD) in relation to the current situational challenges and needs. It should always be remembered that the ultimate goal of CPD is providing optimum patient care and achieving best healthcare outcomes either in customary practice or in exceptionally challenging environments.

## A viral outbreak and a CPD dilemma

Concomitant with the COVID-19 outbreak, a medical education dilemma has emerged. Undergraduate (UG) and postgraduate (PG) medical education are significantly impacted by COVID-19 with a considerable number of published reviews. CPD is no different. However, less attention is paid to CPD due to a number of reasons, one reason being the format of CPD programs that doesn’t feature an academic structure as compared to UG and PG medical education. In addition, the subtle short-term consequences of interrupted CPD programs on healthcare delivery, doesn’t deem it vital to consider CPD strategy modifications by different stakeholders. Furthermore, jurisdictions relaxed the CPD requirements for re-licensing and re-certifications. Hence, CPD is more vulnerable to disruptions with the risk of mid and long-term detrimental impact on HCP performance and ultimately patient care.

The Coronavirus pandemic is an opportunity to carefully consider the following questions in order to ensure the appropriateness and sustainability of any required or implemented changes to CPD systems.

• How can CPD be strategized and operationalized in challenging times while acting in the best interests of patients and people?
• How can CPD be regulated in a transparent and accountable way without overburdening healthcare systems or interrupting the delivery of patient-care services?
• How can CPD be relevant to the needs of an overwhelmed healthcare system and overworked HCP?
• How can we ensure that the introduced modifications are effective?

These questions are meant to stimulate a productive discussion of the topic and to encourage further research but it is not intended or expected to fully answer them in this paper.

## CPD policies and regulation in light of the novel Coronavirus

The COVID-19 epidemic continues to cause disruption to healthcare organizations and services across the globe. Regulatory authorities and healthcare jurisdictions acknowledge the considerable pressure affecting members of healthcare teams whose current priority is to adapt to new clinical situations and areas of clinical practice. They appreciate that administration and accreditation of CPD activities as well as credit submissions by HCP are unlikely to be a current priority.

Coping with Coronavirus, regulators have introduced new relaxed policies and procedures to ease the burden of CPD requirements in appreciation for the noble role played by HCP to provide clinical services and support patients and families during the pandemic ( Royal College of Physicians of Edinburgh, 2020). Supportive statements as well as resources, evidence and profession specific guidance are released by regulators across the globe ( General Pharmaceutical Council, 2020). Alterations and CPD operational changes that are introduced by regulatory authorities include but are not limited to; limitless e-Learning credits, free and reduced subscription rates and flexible category-related CPD requirements ( Royal College of Physicians of Edinburgh, 2020).

## A suggested framework for CPD management during challenging times

Considering the factors relevant to the environment in which the HCP are working at the present times as well as the relevant available information about resources, healthcare systems and HCP needs and priorities; CPD operational procedures should be revised. It is time to consider a novel and unencumbered approach to planning and delivering CPD. At these times, the first concern of HCP will be the care of their patients and people who use health care services. In these highly challenging circumstances, CPD providers may need to depart from established procedures in order to continue to deliver their mission.

In this section, I propose a framework that might help address the emerging challenges in the provision of CPD. The framework encompasses six integral components pertaining to the core principles of medical education, listed as follows:

1. **R**elevance
2. **R**esource oriented
3. **R**esponsive to needs
4. **R**egulated
5. **R**edesigned educational modalities
6. **R**evised evaluation tools and techniques

### 1. Relevant CPD programs

The notion of relevanceimplies that CPD equips HCP by the knowledge and skills to enhance their capability of solving problems that arise in practice and to respond to the community health needs ( Quintero, 2014). CPD learning objectives should focus on finding solutions to real problems with which HCP are confronted in their professional or personal lives ( Knowles, Holton and Swanson, 1998). The concept that healthcare education ought to be oriented to the health needs of the community is extensively discussed in the literature ( Chastonay *et al.,* 1996).

Without doubt, medical knowledge especially clinical practice guidelines, and management protocols are of crucial importance at all times. However, relevance implies CPD providers are mindful of the utmost importance of practice and system-based competences in the complex situation we are currently facing.

### 2. Resource oriented

It is understandable and logical that at the present times healthcare resources are primarily directed to patient care. CPD financing is not considered as a priority in the current situation. CPD funding policies are revisited, mostly leading to resource limitation. This requires CPD providers to consider alternative approaches for securing budgets that needs to be carefully managed. In terms of human resource management, this would be a perfect time for more contribution of medical personnel who are not directly involved in patient care, e.g., academicians and researchers. Experts and educators from non-medical fields would also be of great help in supporting CPD activities addressing personal, communication and leadership skills.

### 3. Responsive to needs

A dynamic CPD model-responsive to the needs and feedback of stakeholders is an essential component of a well-managed and effective CPD system. A responsive CPD system must address the needs of individual HCP, the community they serve and the organizations within which they work, as well as the broader healthcare system and national policy-making institutions. The current time challenges leadership to translate this notion to action. This imposes some revisions of the traditional models used for design and delivery of CPD with special attention to the format, accessibility and convenience for HCP.

### 4. Regulated

Accreditation continues to be crucial for assuring the quality of CPD. At these times, accreditation authorities are challenged by a number of emerging issues e.g. reviewers’ availability, site visits, in addition to the fact that most healthcare systems and organizations are questioning the priority level of the accreditation process amidst the current healthcare crises. This is further aggravated by alterations made by licensing jurisdictions in relation to relaxing their CPD requirements, a step that can temporarily conceal the accreditation mission and value. As a judicious response to those challenges, accreditation bodies need to consider simplified processes, including briefer documentation and more flexible procedures, thus, reducing the burden on the overwhelmed healthcare systems. For accreditation officials, this is not an easy job. Simplification of processes should not compromise the interpretation of and compliance to quality standards neither it should affect the monitoring role of accrediting bodies. The established concepts of substantial equivalence ( McMahon *et al.*, 2016; Accreditation Council for Continuing Medical Education, 2019) and joint accreditation (Joint Accreditation for Interprofessional Continuing Education, 2019) are good examples and worthwhile applications for a simplified and less bureaucratic quality assurance process while sustaining the core principles of accreditation.

### 5. Redesigned educational modalities

The competition between the busy nature of service delivery and securing time for CPD was often cited ( Schostak *et al.*, 2010). It is irrational to try to address this issue at the time being. Alternatively, other learning modalities that do not require protected time nor compete with healthcare provision should be revisited e.g., learner-led education, networking and peer review of practice. Considering the fact that CPD can take place in the workplace, it is useful to consider the positive features within the dynamic relation between CPD and the complexities of the clinical settings where educational opportunities and service delivery requirements interact. Learner-led CPD is an extremely effective educational approach that encourages engagement and acknowledges professionalism ( Schostak *et al.*, 2010). Collaborative and peer group learning are modalities that provide HCP with ways of comparing the quality of their practice and learning in their work place ( Bostrom *et al.*, 2008). Besides being practical solutions for currently encountered issues, the above-mentioned modalities are likely to embrace and enhance the principles of individualized learning, team based and interprofessional education.

### 6. Revised Evaluation Tools and Techniques

Evaluation of CPD continues to be the most challenging step in the CPD process. Modifications mandated by the current situation requires refinement of the traditional evaluation techniques. One question to be answered is: How can CPD in a busy workplace be systematically assessed in terms of the quality of the educational experience and its actual effectiveness? Educators are invited to study and design tools that are realistic and practical to measure the efficiency of the currently introduced modalities, formats and procedures aiming to evaluate short term consequences as well as aspire for long term outcome assessment. Data gathered and analyzed from such evaluations can play a significant role in CPD reformation.

## Factors that contribute to the success of the suggested framework

• A committed and involved leadership.
• Effective communication between stakeholders.
• Meaningful and reliable data collection, analysis and reporting.
• Appropriately managed and efficiently utilized resources.
• A knowledgeable and empowered CPD team.
• Optimal employment of technology.

## Conclusion

The management of CPD during challenging times requires the adoption of a tailored, flexible and context-oriented approach by educators and CPD providers. Accreditors are urged to attain the balance between educational quality assurance and simplified procedures. The overwhelming situation highlights the necessity of synergetic integration of CPD principles and accreditation standards across the world in a technique that promotes sharing of learning resources, collaborative engagements of CPD systems and facilitation of CPD globalization.

## Take Home Messages

The following elements are considered instrumental in achieving a CPD system that adapts to changes and responds to challenges:

• Dynamic system - responsive to the needs and feedback.
• A more permissive system that embraces creativity and innovations.
• Cultural shift to valuing learning and learners over process.
• Data management.
• Efficient resource utilization.
• Focus on performance of health care teams.
• Meaningful relationships including stakeholder engagement.

## Notes On Contributors

**Dr Samar Aboulsoud** is a CPD enthusiast and an advocate of advancing the quality of healthcare outcomes. She is an associate professor of medicine at Cairo University with specific expertise in educational leadership, accreditation and regulation of healthcare systems. She is specially interested in knowledge management and its effective translation to healthcare practice. Dr Aboulsoud has worked in several capacities in administration, education, quality and patient care in academic, corporate, government, and not-for-profit organizations with a long track record of project execution and globally recognized accomplishments. Among her achievements is the construction of an internationally celebrated CPD accreditation system for an entire nation. Dr Aboulsoud is the chair of the CPD committee of the AMEE. She is a member of GAME board of directors, a member of the Academy of Medical Educators, and a fellow of the Higher Education Academy.

ORCID ID: https://orcid.org/0000-0002-8217-9547

## Declarations

The author has declared that there are no conflicts of interest.

## Ethics Statement

Ethical approval is not applicable nor required for this article.

## External Funding

This article has not had any External Funding

## References

[ref1] Accreditation Council for Continuing Medical Education . (2019) ACCME’s Substantial Equivalency program. Available at: https://accme.org/sites/default/files/2019-03/386_20190306_ACCME_Substantial_Equivalency_Framework.pdf(Accessed: 13/11/2020).

[ref2] Bostrom R. P. Gupta S. and Hill J. R. (2008) Peer-to-peer technology in collaborative learning networks: applications and research issues. International Journal of Knowledge and Learning. 4(1), p.36. 10.1504/IJKL.2008.019736

[ref3] Chastonay P. Brenner E. Peel S. and Guilbert J. (1996) The need for more efficacy and relevance in medical education. Medical Education. 30(4), pp.235–238. 10.1111/j.1365-2923.1996.tb00823.x 8949533

[ref4] General Pharmaceutical Council . (2020) Joint statement from the Chief Executives of statutory regulators of healthcare professionals. Available at: https://www.pharmacyregulation.org/sites/default/files/conflicts_of_interest_joint_statement.pdf( Accessed: 12/11/2020).

[ref5] Joint accreditation interprofessional education (2019). Joint accreditation. Available at: https://www.jointaccreditation.org/sites/default/files/20191204_JA_Flyer.pdf( Accessed: 13/11/2020).

[ref6] Knowles M. S. Holton E. F. and Swanson R. A. (1998) The adult learner: a neglected species. Houston: Gulf Pub.

[ref7] McMahon G. T. Aboulsoud S. Gordon J. Mckenna M. (2016) Evolving Alignment in International Continuing Professional Development Accreditation. Journal of Continuing Education in the Health Professions. 36(1): pp.22–26. 10.1097/CEH.0000000000000075 27584065

[ref8] Quintero G. A. (2014) Medical education and the healthcare system - why does the curriculum need to be reformed? BMC Medicine. 12(1), p.213. 10.1186/s12916-014-0213-3 25387484 PMC4228189

[ref9] Royal College of Physicians of Edinburgh .(2020) CPD requirements and the impact of COVID-19. Available at: https://www.rcpe.ac.uk/sites/default/files/files/cpd_diary_statement_updated_for_covid-19_03_04_2020.pdf( Accessed: 13/11/2020).

[ref10] Schostak J. Davis M. Hanson J. Schostak J. (2010) Effectiveness of Continuing Professional Development’ project: A summary of findings. Medical Teacher. 32(7), pp.586–592. 10.3109/0142159X.2010.489129 20653382

