# Supplementary material for: We “R” capable of leading CPD in challenging times: A suggested framework for CPD management during COVID-19 and beyond
Source: MedEdPublish (2016). 2021 Jan 11;10:2. [Version 1] doi: 10.15694/mep.2021.000002.1 (PMC10917084; doi:10.15694/mep.2021.000002.1)
Supplement: Peer review history

# Reviewer report: Reviewer response for version 1

**DOI:** 10.21956/mep.18861.r26901

**Published:** 2021-03-12

**Authors:** Barbara Jennings

**Affiliations:** 1 Norwich Medical School

This review has been migrated. The reviewer awarded 4 stars out of 5 I really enjoyed reading this article and agree with the author’s personal view and the conclusions presented. It will be of use to curriculum leads for faculty development as they consider the impact of the pandemic and the need for efficient and effective policies to support professional development. We have all realised how much unnecessary resource has been spent on unsustainable teaching events or processes linked to regulation that may serve an internal bureaucracy more than reliable quality standards. Covid-19 has forced us to notice many weaknesses & cracks in prevailing systems and to seek new approaches.Responsive and redesigned educational models are required e.g. with just-in-time training; & creative and agile technology enhanced options that are aligned to relevant outcomes.The structure of this article was very good for presenting coherent arguments in an engaging way about these issues. I think the framework will be just as relevant to CPD during the ongoing expansion of clinical and healthcare professional training (which we are all in need of across the globe) as it is for the current crisis.

Reviewer Expertise:

NA

No decision status is available

# Reviewer report: Reviewer response for version 1

**DOI:** 10.21956/mep.18861.r26900

**Published:** 2021-01-21

**Authors:** Annwyne Houldsworth

**Affiliations:** 1 HECL

This review has been migrated. The reviewer awarded 4 stars out of 5 I would like to congratulate the author on the paper entitled, ‘We “R” capable of leading continued professional development (CPD) in challenging times: A suggested framework for CPD management during COVID-19 and beyond’ as it is extremely well written with respect to grammar and spelling and has a very witty punning title, so appropriate for the current global situation.I appreciate the six R’s concept in this paper as a tool for innovation. We certainly require some innovative methods to train, maintain, support and celebrate our (HCP) care professionals during this pandemic.I particularly like the innovative suggestion that the author recognises the need for active learning at work to support continued professional. The simple act of staff sharing their experiences and the impact their work has had on themselves and their patients would be very useful CPD in itself.The gathering of essential information through data collection is an important suggestion as this could well be overlooked in the overwhelming conditions that the COVID 19 pandemic has brought to hospitals and, in particular, intensive care departments.The use of online facilities to deliver CPD is essential at the moment but the author could include the importance of digital/online conferencing and providing facilitators to deliver the material, which could enable those engaging in the CPD to interact with each other and even split into smaller groups to discuss questions about the subject being addressed in the syllabus. Sharing of resources would be also be relevant and achieved by the vertical transmission of knowledge and experiences from those professionals taking part in the session when they share their own experiencing relevant to the topic. This could also be responsive to current needs and regulated by the course providers, facilitators and feedback from those taking part in the session. Assessors could monitor external observation of the sessions at regular times.The author’s reference to rethinking and redesigning HDP CPD curriculum, syllabus and delivery is so important at the moment.CPD in interprofessional teamwork has never been so important as it is today in the very strained ICU departments of hospitals with long term very sick patients.Some actual examples to demonstrate some of the text would add a helpful narrative to the issues addressed.I wish to thank you for drawing attention to this very important issue.

Reviewer Expertise:

NA

No decision status is available

# Reviewer report: Reviewer response for version 1

**DOI:** 10.21956/mep.18861.r26899

**Published:** 2021-01-17

**Authors:** Helena Filipe

**Affiliations:** 1 Hospital of the Armed Forces/PL-EMGFA

This review has been migrated. The reviewer awarded 4 stars out of 5 Thank you for the opportunity to review this article.The Author elaborates on the effects of the pandemic on CPD emphasising its variable academic structure and regulation requirements and proposes a framework to tackle emerging challenges. Nicely entitled, the article invites us to find more about the possible meaning(s) for the “r” and calls for the CPD community’s collective thinking. Anchoring the willingness to take the disruptive pandemic situation as a challenging opportunity, a feeling of curiosity, adaption and growth perspires across the article.While recognising classical components of good CPD in the definitions featured by the six “r”s we discover how the Author proposes to build on them to effectively adapt and evolve. A note of remark for considering team competence (multidisciplinary and interprofessional).Pivoting to online learning strategies will further innovation in CPD activities and systems’ design and generate roles’ reorganisation. Perhaps some illustrating examples coupled with the descriptions of each framework component could further encouragement. (Mack and Filipe, 2020) The pandemic has prompted a paradigm shift into the digital world accelerating the need to innovate and value creative learning modalities. (Mealey, 2019; Price and Campbell, 2020) e virtual communities of practice curated by faculty expert in scholarly teaching and developing scholarship of teaching are increasingly receiving widespread attention. (Chan and Ankel, 2021) Dynamic, effective, evidence consistent and creative yes, permissive not so.Despite understanding the humanistic value argued in “Cultural shift to valuing learning and learners over process” we would highlight the importance of the context intangibles of the learning process for a whole program evaluation. (Frye and Hemmer, 2012)Practice and learning are increasingly intertwined at the point of care as the readiness to learn on the move about illness beyond knowing the disease.How can this type of learning be accredited and demonstrable? How much of a role will organisations have in promoting these social practice-based learning spaces and determine the quality of CPD and consequently education and patient outcomes?Should this be a requirement as a quality component in organisations’ accreditation?At the forefront of other medical education stages, will CPD continuing progressive atomisation dimmer classic events?What competencies should CPD educators need to master, to create demonstrable and amenable to accreditation learning experiences to embed into the HCPs daily working? HCPs are urged into self-determined learning (Blaschke, 2012) and master a set of competency domains (Campbell et al, 2010) Through critical thinking and self-reflection, the adaptive learner (Cutrer et al, 2017) becomes capable to apply competencies in novel situations. Technology can be viewed as a personal partner in bringing, finding and storing information and finding people sharing similar goals. Networking constructive professional relationships, lifelong learners and educators become peers and co-build communities of practice that can work as supportive and trustful personal learning environments. (Siemens, 2005)An exciting progression is surely on its way. Congratulations to the Author for the initiative and the invitation to reflect on the CPD realm now and beyond.

Reviewer Expertise:

NA

No decision status is available
